# Sodalis ligni Strain 159R Isolated from an Anaerobic Lignin-Degrading Consortium

**DOI:** 10.1128/spectrum.02346-21

**Published:** 2022-05-17

**Authors:** Gina Chaput, Jacob Ford, Lani DeDiego, Achala Narayanan, Wing Yin Tam, Meghan Whalen, Marcel Huntemann, Alicia Clum, Alex Spunde, Manoj Pillay, Krishnaveni Palaniappan, Neha Varghese, Natalia Mikhailova, I-Min Chen, Dimitrios Stamatis, T. B. K Reddy, Ronan O’Malley, Chris Daum, Nicole Shapiro, Natalia Ivanova, Nikos C. Kyrpides, Tanja Woyke, Tijana Glavina del Rio, Kristen M. DeAngelis

**Affiliations:** a Department of Microbiology, University of Massachusetts–Amherst, Amherst, Massachusetts, USA; b United States Department of Energy Joint Genome Institute, Berkeley, California, USA; University of Southern Denmark

**Keywords:** endosymbionts, aromatic metabolism, lignocellulosic biofuel, anaerobic catabolic pathways, aromatic compounds

## Abstract

Novel bacterial isolates with the capabilities of lignin depolymerization, catabolism, or both, could be pertinent to lignocellulosic biofuel applications. In this study, we aimed to identify anaerobic bacteria that could address the economic challenges faced with microbial-mediated biotechnologies, such as the need for aeration and mixing. Using a consortium seeded from temperate forest soil and enriched under anoxic conditions with organosolv lignin as the sole carbon source, we successfully isolated a novel bacterium, designated 159R. Based on the 16S rRNA gene, the isolate belongs to the genus *Sodalis* in the family *Bruguierivoracaceae*. Whole-genome sequencing revealed a genome size of 6.38 Mbp and a GC content of 55 mol%. To resolve the phylogenetic position of 159R, its phylogeny was reconstructed using (i) 16S rRNA genes of its closest relatives, (ii) multilocus sequence analysis (MLSA) of 100 genes, (iii) 49 clusters of orthologous groups (COG) domains, and (iv) 400 conserved proteins. Isolate 159R was closely related to the deadwood associated *Sodalis* guild rather than the tsetse fly and other insect endosymbiont guilds. Estimated genome-sequence-based digital DNA-DNA hybridization (dDDH), genome percentage of conserved proteins (POCP), and an alignment analysis between 159R and the *Sodalis* clade species further supported that isolate 159R was part of the *Sodalis* genus and a strain of Sodalis ligni. We proposed the name Sodalis ligni str. 159R (=DSM 110549 = ATCC TSD-177).

**IMPORTANCE** Currently, in the paper industry, paper mill pulping relies on unsustainable and costly processes to remove lignin from lignocellulosic material. A greener approach is biopulping, which uses microbes and their enzymes to break down lignin. However, there are limitations to biopulping that prevent it from outcompeting other pulping processes, such as requiring constant aeration and mixing. Anaerobic bacteria are a promising alternative source for consolidated depolymerization of lignin and its conversion to valuable by-products. We presented Sodalis ligni str. 159R and its characteristics as another example of potential mechanisms that can be developed for lignocellulosic applications.

## INTRODUCTION

In the family *Bruguierivoracaceae*, the genus *Sodalis* can be grouped generally into three guilds: deadwood associated isolates, endosymbionts associated with the tsetse fly, and endosymbionts of other insects, such as long-horned beetles, louse flies, and bees (1–4). The genus was established by Dale and Maudlin (5) with the description of species S. glossinidius strain M1, which was the first isolated insect secondary endosymbiont. Insect secondary endosymbionts are recently established symbiotic associations that can be horizontally or vertically transmitted as well as introduced to the host by the environment (6). There is evidence that certain insect hosts may also serve as vectors for the transmission of *Sodalis* to alternative hosts, such as plants (7). Sodalis endosymbionts and allied species, known as the Sodalis-allied clade, contain genomes that are small and degenerated, having lost most of the gene inventory (8, 9). Because many of the *Sodalis* species are speculated to be more recently acquired symbionts, there is a unique opportunity to understand how these associations evolve (9–11). However, resolving the evolutionary relationships of *Sodalis* is difficult due to *Sodalis* genomes containing pseudogenes, mobile DNA, gene rearrangements, duplications, and deletions (9, 12).

There is a total of three free-living *Sodalis* members identified to date: S. praecaptivus (13), Sodalis ligni dw23, and *Sodalis* sp. dw96 (1). S. praecaptivus was isolated from an infected wound of a person whose hand had been impaled by a dead crab apple tree branch, suggesting that S. praecaptivus was either a pathogen or saprophyte residing on the bark or woody tissue of the plant (14). Sodalis ligni dw23 and *Sodalis* sp. dw96 were both isolated from decomposing deadwood (1). Bacteria isolated from decomposing wood or plant litter on the soil surface are part of a much larger microbial community that includes members that depolymerize lignin to access cellulose or hemicellulose components of plant material, that consume the lignin-derived monomers, or can do both functions (15). Efforts to understand the mechanisms of how these organisms use lignocellulose as a raw material may accelerate the development of microbial applications for paper and biofuel manufacturers (16, 17).

Here, we described a novel, free-living Sodalis ligni strain isolated from temperate forest soils (Petersham, MA, USA), and propose the species name Sodalis ligni strain 159R. Using the sequenced genome of strain 159R, we aimed to enhance our understanding of endosymbiont evolution and the anaerobic decomposition of lignin. Having one of the largest genomes to date within *Sodalis*, this strain was more diverse in its metabolic capabilities compared to S. praecaptivus and its endosymbiotic relatives, including the genetic potential to catabolize plant-derived aromatics like vanillate and catechol.

## RESULTS AND DISCUSSION

### Isolation and 16S rRNA gene phylogeny.

Strain 159R did not display depolymerizing activity for both malachite green and Congo red dyes. However, its high tolerance for lignin concentrations, and its persistence through 7 serial transfers in the consortia, indicated that strain 159R may catabolize lignin-derived monomers within the organosolv enriched microbial community under anoxic conditions (18). Therefore, we chose to investigate this organism further.

Based on 16S rRNA gene sequence homology, strain 159R was 96.79% identical to Sodalis praecaptivus HS, 96.38% identical to Sodalis glossinidius, and 95.97% identical to Biostraticola tofi, which was a close relative to the *Sodalis* clade. Using NCBI BLASTn (19), we also compared the 16S rRNA gene of 159R to Sodalis ligni dw23, which was 97.35% identical, and close relative, Bruguierivorax albus, which was 96.05% identical. The 16S rRNA gene sequence for *Sodalis* sp. dw96 was not annotated and, therefore, was not included in this analysis. Because strain 159R was less than 97% identical in rRNA gene sequence to all known relatives other than dw23, with the latter still being under the conservative threshold of 99% (20), we considered this evidence that strain 159R may be a novel *Sodalis* species (21). It is important to note that strain 159R had a 99% similarity to an isolate deemed *Yersinia* sp. KM16 (22), which was 96.2% similar to Yersinia ruckerii. Further investigation of KM16’s 16S rRNA gene found that it was 97.84% similar to S. praecaptivus HS, supporting that our isolate, and likely KM16, were in the *Sodalis* genus rather than *Yersinia*. KM16 did not have its genome sequenced and, therefore, was not used for further analyses.

**FIG 1:**
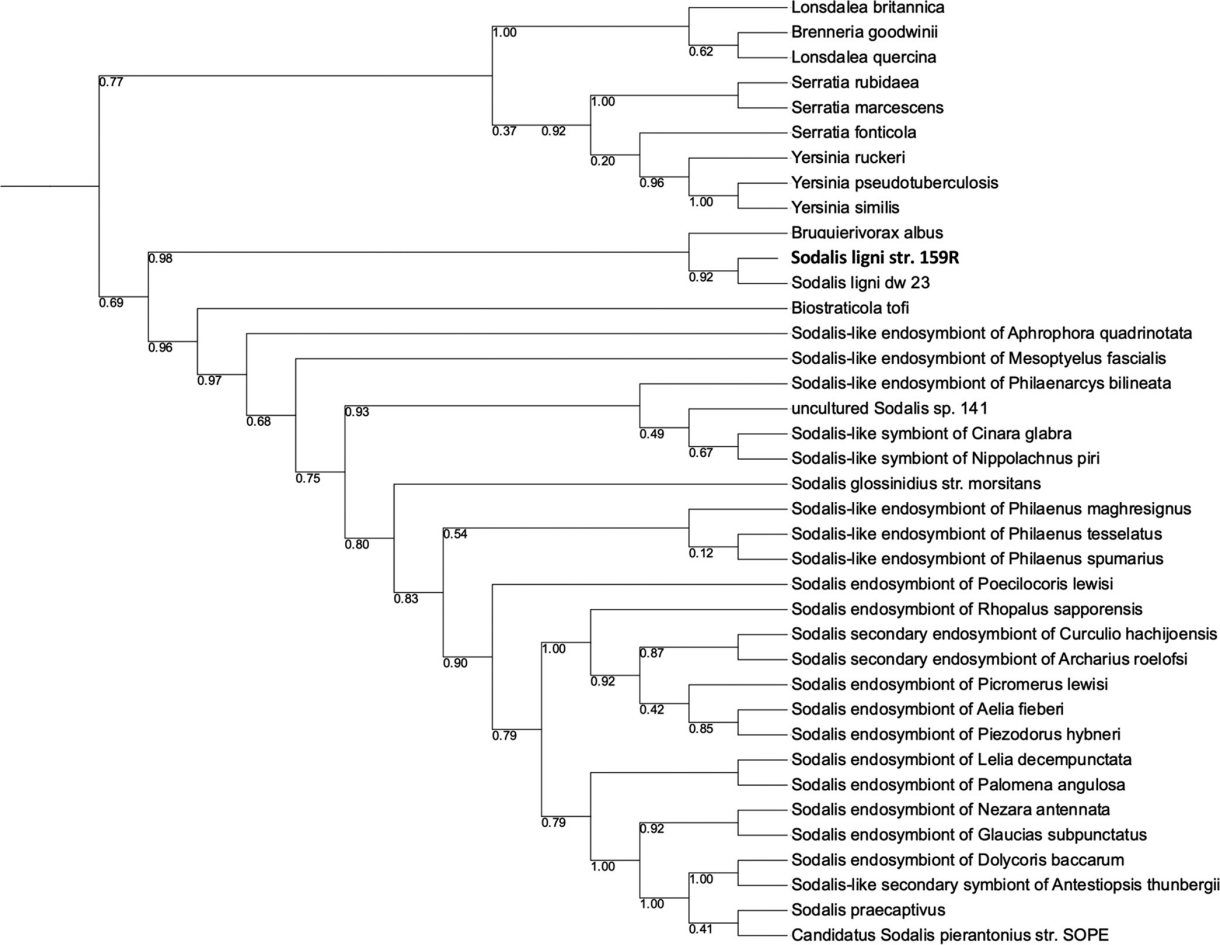
Reconstruction of the phylogenetic position of strain 159R based on 16S rRNA gene. Trees are presented as maximum-likelihood trees with bootstrap values. The tree was rooted in an outlier, Methanocaldococcus jannaschii (not shown in the tree).

To explore the initial phylogenetic position of 159R, we used 16S rRNA gene sequences to construct a phylogenetic tree (Fig. 1). While *Serratia* and *Yersinia* species formed monophyletic clades, the *Sodalis* clade was a polytomy and had less resolution. The *Sodalis* clade includes close relatives B. albus and B. tofi between the deadwood guild and the two endosymbiont guilds. Adeolu et al. (23) described a similar instance where the *Sodalis* members branched differently when comparing a 16S rRNA gene-based tree to a multilocus sequence tree as well as a genome-based tree, suggesting that the endosymbiotic adaptations were the cause of this discrepancy. Because strain 159R had the potential for anaerobic aromatic metabolism and due to its provenance, its genome was chosen for sequencing to gain a better understanding of its potential role in the soil microbiome.

### Genomic features and phylogeny.

The final draft assembly of strain 159R contained 1 contig in 1 scaffold, totaling 6,384,591 bp in size. The input read coverage was 45.4× with a G+C content of 54.98 mol%. One chromosomal origin of replication, located at 3384 kb, was identified with the oriloc function from the seqnir R package (Fig. S2) (24). Based on average nucleotide identity (ANI), strain 159R was 76 to 77% similar to the two endosymbionts *Sodalis* guilds (Table 1), well below the accepted 95 to 96% species threshold (25). Strain 159R has a dDDH value less than the 70% species boundary compared to all endosymbiont genomes of the *Sodalis*-allied clade (Table 1) (25). However, comparing 159R to members of the deadwood guild, S. ligni dw23, and *Sodalis* sp. dw96, dw96 was more distantly related (ANI 87.4%, dDDH 34.6%) whereas there was evidence that 159R was the same species as S. ligni dw23 (ANI 98.24%, dDDH 85.9%) (Table 1).

**TABLE 1:** Genome size, average nucleotide identity (ANI), average amino acid identity (AAI), digital DNA-DNA hybridization (dDDH), and percentage of conserved proteins (POCP) estimates comparing Sodalis ligni str. 159R (6.38Mbp) to the *Sodalis*-allied clade member taxa and closest relatives based on 16S rRNA genes

Organism	Genome size (Mb)	ANI %	AAI %	dDDH estimate % (GLM-Based)	POCP %
Sodalis ligni dw23	6.44	98.24	97.82	85.90	85.90
*Sodalis* sp. dw96	5.9	87.40	90.38^*a*^	34.60	82.69^*a*^
Sodalis praecaptivus HS	5.15	78.97	72.55	21.50	63.68
Biostraticola tofi DSM 19580	4.29	78.71	73.30	20.80	61.87
Bruguierivorax albus BGMRC 2031	5.66	77.69	77.76	22.00	70.35
Candidatus Sodalis pierantonius SOPE	4.51	79.10	73.23	21.40	51.27
Candidatus Sodalis sp. SoCistrobi 3249	3.06	79.06	74.60	20.90	48.82
*Sodalis* sp. TME1	3.41	79.35	73.70	22.00	42.65
Sodalis glossinidius mositans	4.29	79.22	71.37	22.00	41.95
*Sodalis*-like endosymbiont of Proechinophthirus fluctus	2.17	78.94	68.41	22.30	29.77
*Sodalis*-like symbiont of Philaenus spumarius PSPU	1.38	78.96	74.97	21.80	29.13

a Values may be unreliable due to incomplete gene annotation publicly available.

To further explore whether strain 159R was the same species as S. ligni dw23, the percentage of conserved proteins (POCP) was calculated comparing strain 159R to *Sodalis* members as well as close relatives B. albus and B. tofi (26, 27). POCP estimates genus demarcation between two organisms based on proteins that were shared (26). If the POCP was greater than or equal to 50%, then the organisms were considered to be within the same genus. Strain 159R had a POCP of 51.27% for Candidatus ‘Sodalis pierantonius SOPE’, a POCP of 61.87% for B. tofi, and a POCP of 63.68% for S. praecaptivus HS. All other POCP values were less than 50% when comparing strain 159R to other *Sodalis* members (Table 1). However, these low POCP values could be explained by genome degeneration of the *Sodalis* endosymbionts (26). POCP results supported that strain 159R was within the same genus as S. praecaptivus HS as well as Candidatus Sodalis pierantonius SOPE, which was one of S. praecaptivus’ closest relatives and similar in genome size. We then compared 159R to the recently published S. ligni dw23 and *Sodalis* sp. dw96 (Table 1). However, dw96 was missing annotations, such as 16S rRNA, suggesting that the comparison may be inadequate. Regardless, POCP values for 159R were 85.9% and 82.69% compared to dw23 and dw96, respectively. These results further supported that 159R and S. ligni dw23 were strains of the same species.

**FIG 2:**
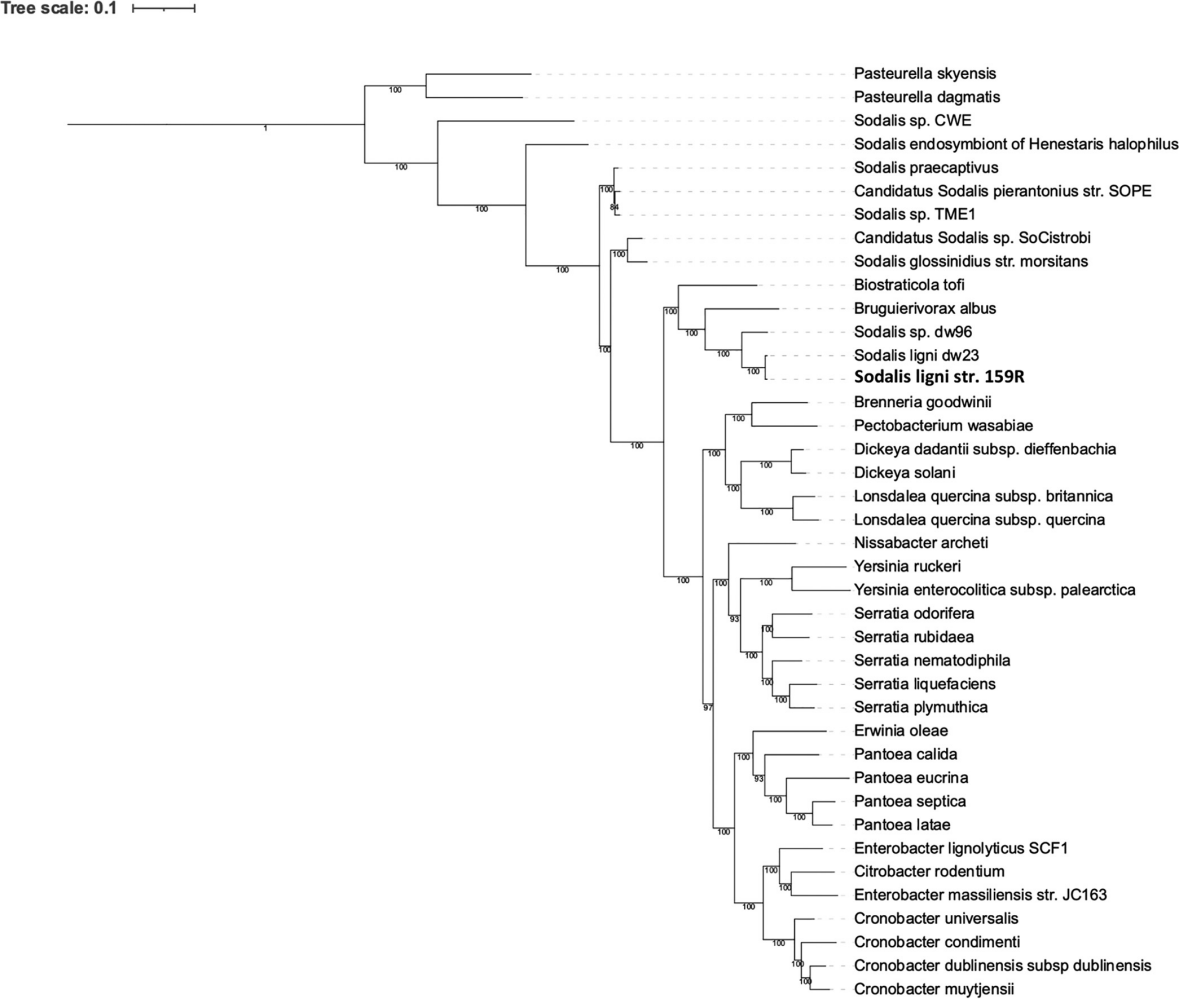
Reconstruction of the phylogenetic position of Sodalis ligni str. 159R-based multi-locus sequence analysis (MLSA) via autoMLST. Trees are presented as maximum-likelihood trees with bootstrap values.

**FIG 3:**
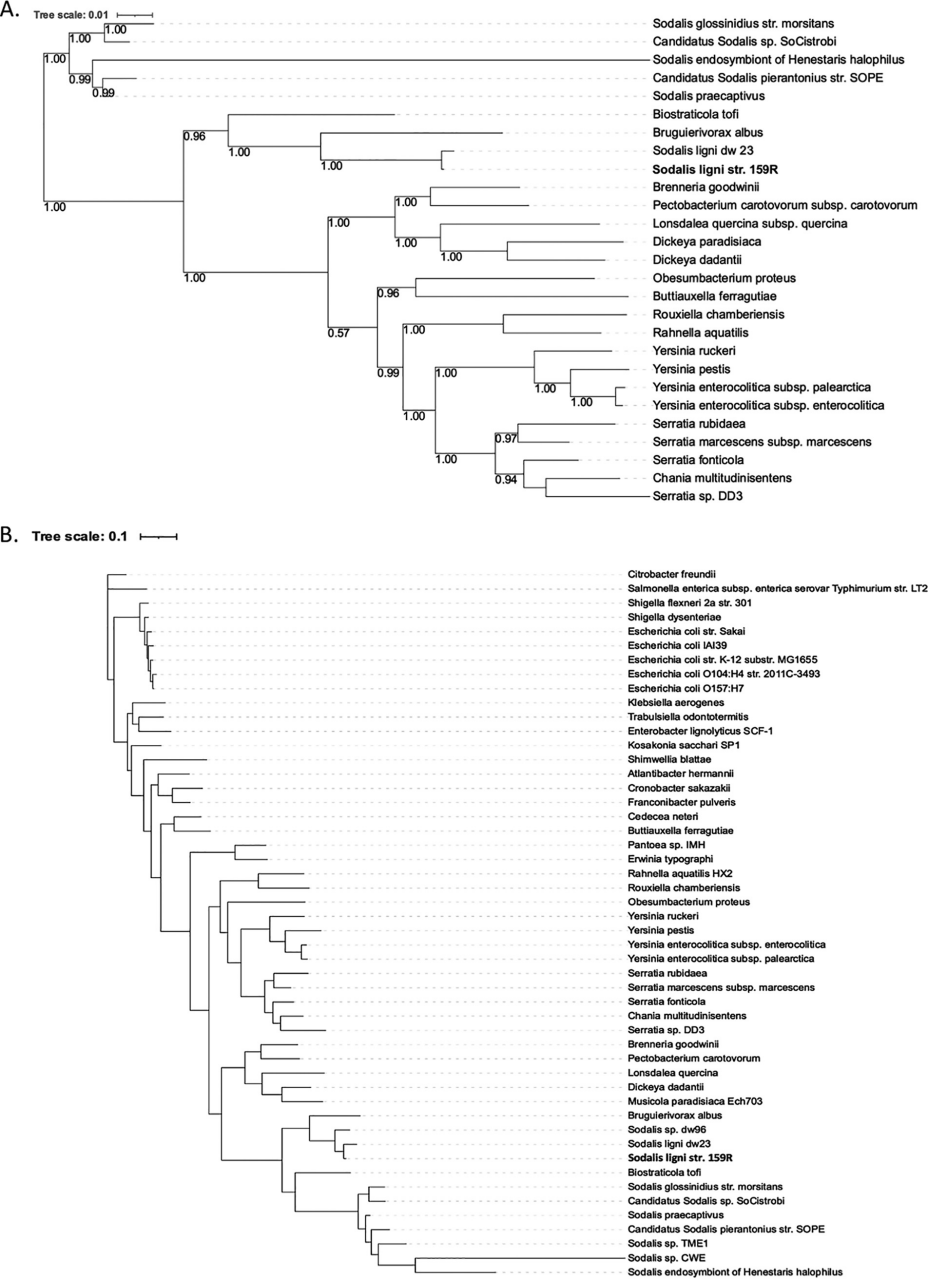
Reconstruction of the phylogenetic position of strain 159R based on (A) alignment similarity for a subset of 49 COG domains using KBase insert genome into species tree and (B) 400 conserved protein sequences using PhyloPhlan. The KBase insert genome species tree is presented as maximum-likelihood trees with bootstrap values.

To gain resolution of the phylogenetic position of strain 159R, phylogenetic trees were constructed using three different methods: (i) multilocus sequence analysis (MLSA) of 100 genes (Fig. 2), (ii) 49 clusters of orthologous groups (COG) domains (Fig. 3A), and (iii) 400 conserved proteins (Fig. 3B). With strong support from all three trees, *Sodalis* sp. strain 159R was positioned as a basal member to the *Sodalis*-allied clade, which contained the endosymbiont guilds, and was grouped with *Sodalis* deadwood guild members, S. ligni dw23 and *Sodalis* sp. dw96. Strain 159R was also grouped with dw23 in every tree, further supporting that these two bacteria were strains of the same species. However, because strain 159R and other deadwood guild members were also more distantly related to the *Sodalis-*allied clade than Biostraticola tofi and Bruguierivorax albus, strain 159R as well as S. ligni dw23 and *Sodlais* sp. dw96 could represent a novel genus, rather than a species within *Sodalis*.

To explore the evolutionary relationship between strain 159R, B. tofi, and B. albus, we first reviewed the previous characterization of B. tofi. Verbarg et al. (13) made B. tofi a new genus due to the “distant phylogenetic position compared to any other representative of the (*Bruguierivoracaceae*) family and the significant phenotypic differences to its nearest phylogenetic neighbor, Sodalis glossinidius”. The phenotypic differences observed between B. tofi and S. glossinidius morisitans, such as carbon substrate utilization, were most likely due to the smaller genome size of the latter compared to that of its “evolutionary precursor,” S. praecaptivus (8). Therefore, we calculated the POCP between B. tofi and S. praecaptivus to see if B. tofi may phylogenetically fall within the *Sodalis* clade. The POCP was 68.44%, supporting that B. tofi was very similar to the *Sodalis* free-living members and potentially was not a separate genus. However additional comparisons of genomic features and physiology between B. tofi and S. praecaptivus would be needed to confirm this. B. albus was recently published and the authors compared it to strain 159R for 16S rRNA gene similarity and phylogenetic tree position, ANI values, putative gene presence/absence, G+C content, and DDH estimated values. Our analyses and theirs were similar, apart from our dDDH estimated values being lower (22.0% versus 26.8%). Li et al. (28) had compelling evidence, including phenotypic and chemotaxonomic characteristics of B. albus, that supported that B. albus was a distinct species compared to B. tofi. With the discovery of S. ligni dw23 and *Sodalis* sp. 96 after the publication of B. albus, we recommended further investigation of the genomic features and physiology of the *Sodalis* deadwood guild and B. albus.

The genome of strain 159R consisted of 5,684 predicted coding sequences. For energy production, strain 159R had genes encoding aerobic respiration, as well as NarGHI for nitrate reduction, as observed in the core genomes of Sodalis praecaptivus HS, Candidatus Sodalis pierantonius SOPE, and *Sodalis* TME1 (29). Compared to the available 6 genomes of the *Sodalis*-allied clade (Table 1; dw23 and dw96 were not included) as well as B. tofi in the JGI IMG Phylogenetic Profiler, 2,012 genes were unique to *Sodalis* sp. strain 159R, with 1,179 of those unique genes assigned COG IDs (Fig. S3). Tláskal et al. (1) in their pangenome analysis determined that the *Sodalis* deadwood guild shared an average of 1,596 genes from 1,519 gene clusters, which was 28.4% per average genome for each member.

**FIG 4:**
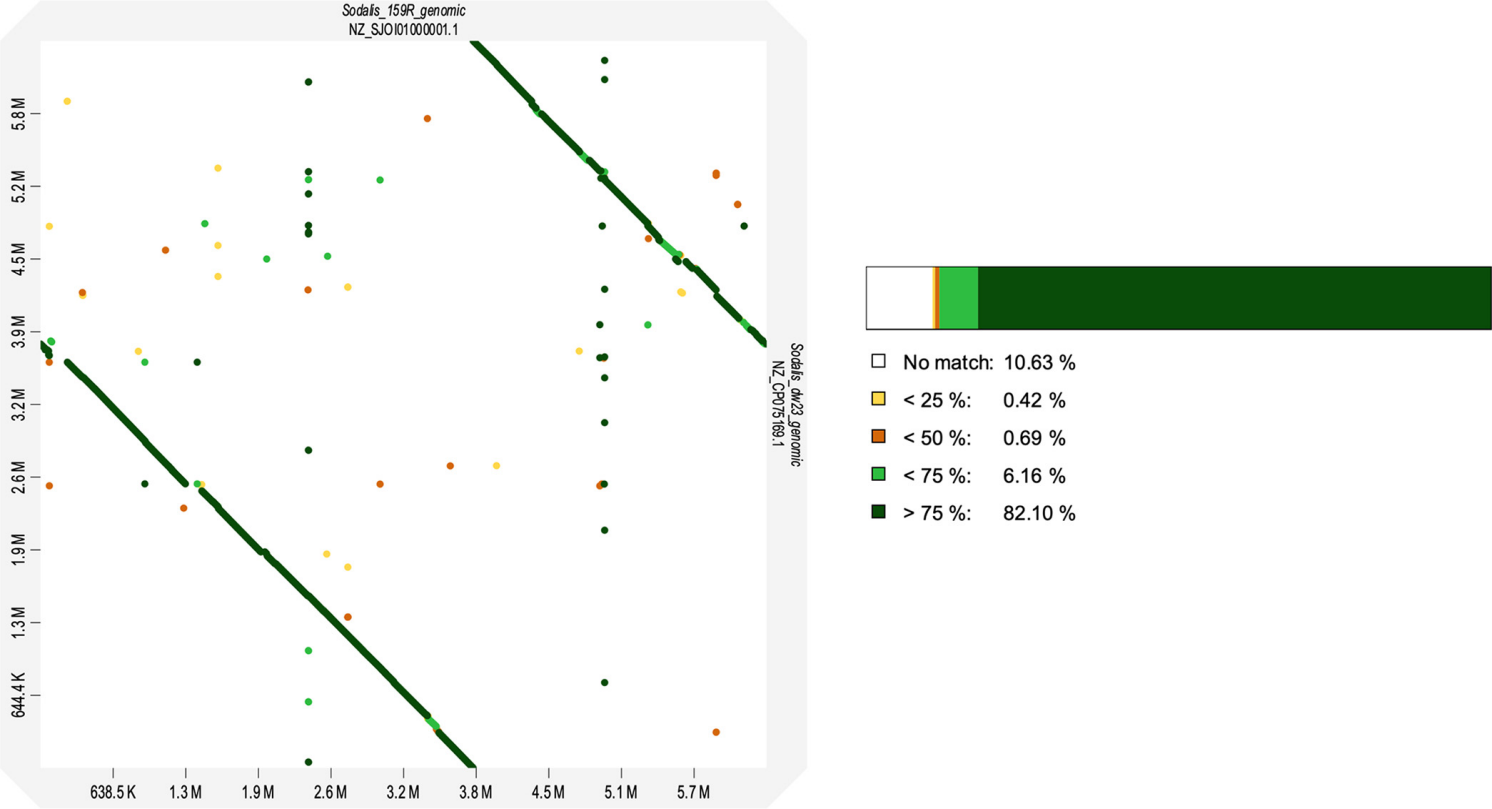
Alignment analysis comparing the chromosome of Sodalis ligni str. 159R to S. ligni dw23 with the online tool, d-Genies, with 159R as the target and dw23 as the query (aligned with Minimap2).

GSAlign analysis determined that there were 3,072 SNVs, 50 insertions, and 120 deletions between the two genomes. Visualization of the genome pairwise alignment corroborated with the high ANI and dDDH estimate values, further supporting that these two isolates were strains within the same species (Fig. 4).

From the Phylogenetic Profiler analysis via Joint Genome Institute Integrated Microbial Genomes (JGI IMG), the largest group of unique genes present in strain 159R were those relating to transcription (222 genes). In comparison to many endosymbionts, free-living organisms tended to be enriched for transcription regulators due to the need to acclimate to ever-changing environmental conditions (30). Similarly, the second largest group of unique genes present in strain 159R were related to carbohydrate transport and metabolism (193 genes). These genes were required to adapt to the varying availability of metabolites found in the soil environment compared to the limited nutrient availability in a host (31). This group of unique genes included those associated with lignocellulose degradation, such as a GH43 family β-xylosidase and a feruloyl esterase, as well as genes for uptake and utilization of aromatic monomers. Enzymes included a 4-hydroxybenzoate transporter-like major facilitator superfamily (MFS) transporter, nine glutathione S-transferases, four catechol 2,3-dioxygenase enzymes, and a salicylate hydroxylase, a vanillate O-demethylase monooxygenase (*vanA*), a vanillate O-demethylase ferredoxin subunit (*vanB*), and a 4-carboxymuconolactone decarboxylase.

### Lignin metabolic potential.

We investigated the genetic potential of strain 159R for anaerobic lignin degradation as well as aromatic catabolism that could be applied toward secondary chemical and biofuel production from lignocellulosic material (32). Enzymes predicted with HMMER to catabolize either benzoyl-CoA or its analogs in strain 159R included homologs to enoyl-CoA hydratase (gene ID 2788604060; E value = 9.4e−55) and hydroxyacyl-CoA dehydrogenase (gene ID 2788603217; E value = 1.9e−55) of the 3-hydroxy benzoyl-CoA pathway. There were no homologs for acyl-hydrolase, which could suggest the presence of an alternative, and possibly novel, enzyme that funnels 3-hydroxy benzoyl-CoA into the central metabolism. Additionally, strain 159R contained a homolog to phloroglucinol reductase (phloroglucinol pathway; gene ID 2788602949, E value = 6.7e−53) as well as homologs to α-resorcylate hydroxylase large subunit (gene ID 2788606053) and small subunit (gene ID 2788606054) with E values 3.3e−48 and 4.5e−44, respectively. Results suggested that 159R was capable of metabolizing aromatics under anoxic conditions and should be further studied to better define the enzymes and pathways that may be present.

In addition to anaerobic aromatic metabolism, enzymes annotated for aerobic aromatic metabolism in the strain 159R genome included 4,5-DOPA dioxygenase extradiol (LigB) as well as homologs with >40% sequence identity to *ligF*, *ligJ*, *ligK*, *ligR*, and *ligV* genes that were also found in the aerobic lignin degrader Sphingomonas paucimobilis SYK-6 (33) (Table 2). Strain 159R also contained genes for the catechol degradation pathway. The genomic potential of lignin degradation and aromatic catabolism under both anaerobic and aerobic conditions are listed in Tables 2 and 3.

**TABLE 2:** Enzymes in Sodalis ligni str. 159R homologous to Sphingomonas paucimobilis SYK-6 genes involved in lignin degradation or metabolism

Enzyme in SYK-6	Bit score	E value	% Identity	159RT gene annotation	Gene ID
Beta-etherase (ligF)	45.8	7e−7	40	Glutathione S-transferase	2788607536
2-keto-4-carboxy-3-hexenedioate hydratase (ligJ)	437	1e−155	60	4-oxalomesaconate hydratase	2788602671
4-carboxy-4-hydroxy-2-oxoadipate aldolase (ligK)	231	9e−78	59	4-carboxy-4-hydroxy-2-oxoadipate aldolase	2788602672
LigR protein (ligR)	273	3e−89	40	Transcriptional regulator/LysR family transcriptional regulator	2788606035
Vanillin dehydrogenase (ligV)	325	3e−107	41	Aldehyde dehydrogenase (NAD+)	2788604477

**TABLE 3:** Putative lignin and aromatic compound degradation/metabolism enzymes in Sodalis ligni str. 159 with general enzyme function descriptions and related references

Gene product	IMG JGI gene ID	Enzyme function	References
4,5-dihydroxy-phenylalanine (DOPA) dioxygenase extradiol	2788604550	Produces betalamic acid by breaking the cyclic ring of DOPA	56
Benzoate membrane transport protein	2788605192	Transports benzoate across the membrane	57
Feruloyl esterase	2788602630	Cleaves the ester-link of xylans and monomeric or dimeric ferulates	58
4-hydroxybenzoate polyprenyltransferase	2788605631	Attaches a polyprenyl side chain to the 4-hydroxybenzoate ring; part of the ubiquinone biosynthesis pathway	59
AAHS family 4-hydroxybenzoate transporter-like MFS transporter	2788601817	Transports 4-hydroxybenzoate and protocatechuate across the membrane	60
p-hydroxybenzoic acid efflux pump subunit AaeAB	2788604686, 2788604687	Efflux pump to alleviate stress from high concentrations of aromatic compounds	61
Vanillate O-demethylase ferredoxin subunit	2788604204, 2788606120	Converts vanillate into protocatechuate	62
Vanillate O-demethylase monooxygenase subunit	2788606119	Converts vanillate into protocatechuate	62
Glutathione S-transferase	788605280, 2788602381, 2788606111, 2788603289, 2788603705, 2788605754, 2788605671, 2788603656, 2788603688, 2788605210, 2788603033, 2788607494, 2788604573, 2788604878, 2788606764, 2788604627	Cleaves the β-aryl ether linkage found in lignin polymers	63
Xylulokinase	2788605934, 2788605929, 2788605827, 2788606718, 2788603328, 2788604167, 2788604787, 2788606042	Converts d-xylose to d-xylose 5-phosphate	64
Alpha-d-xyloside xylohydrolase	2788605868, 2788606010	Releases d-xylose from nonreducing ends of short xylooligosaccharides	65
GH43 family beta-xylosidase	2788606510	Hydrolyzes short xylooligomers into single xylose units	66
Xylose isomerase, xylose isomerase-like TIM barrel protein	2788602980, 2788602989, 2788603070	Catalyzes the interconversion of d-xylose and d-xylulose	67
2-keto-4-pentenoate hydratase/2-oxohepta-3-ene-1,7-dioic acid hydratase in catechol pathway	2788602115, 2788604948, 2788602827, 2788605041	Converts2-hydroxypentadienoic acid to 4-hydroxy-2-ketopentanoic acid	68
4-carboxymuconolactone decarboxylase	2788607467, 2788605411	Part of the protocatechuate branch of the 3-oxoadipate pathway	69
Phenylpropionate dioxygenase-like ring-hydroxylating dioxygenase large terminal subunit	2788605051, 2788604207	Oxidizes aromatic hydrocarbons to cis-arene diols	70
Catechol 2,3-dioxygenase	2788605046, 2788606693, 2788603188, 2788605200, 2788605050, 2788606195	Catalyzes the ring cleavage of catechol and some substituted catechols	71

### Description of Sodalis ligni str. 159R.

Strain 159R formed nonpigmented, opaque circular colonies with shiny surfaces and cells were rod-shaped and Gram-negative. Strain 159R could be grown between 25 and 37°C aerobically, with 30°C being the optimal temperature for growth. At 30°C, optimal growth was observed at a pH of 7. Out of the 95 available substrates tested as carbon sources, strain 159R can assimilate α-d-glucose-1 phosphate, α-d-glucose, α-d-lactose, d-glucose-6-phosphate, d-fructose, d-galactonic acid lactone, d-galactose, d-gluconic acid, d-glucuronic acid, d-mannitol, d-mannose, d-serine, d-sorbitol, d-trehalose, d,l-α-glycerol phosphate, d,l-lactic acid, glycerol, l-aspartic acid, maltose, *N*-acetyl-d-galactosamine, *N-acetyl-d-*glucosamine, pyruvic acid methyl ester, succinic acid, and mono-methyl succinate. Carbon substrate utilization of strain 159R was further compared to available descriptions of S. praecaptivus HS, S. glossinidius, and B. tofi in literature (5, 8, 13) (Table S2).

## MATERIALS AND METHODS

### Isolation and ecology.

Strain 159R was isolated from an anaerobic enrichment culture of temperate forest soil (Harvard Forest, Petersham, MA; 42.54N, 72.18W). Soil samples were taken from plots that had been warmed 5°C above ambient temperature for 23 years at the time of collection, along with consortia derived from control plots that were not heated. Three independently adapted consortia each from the heated plots (H16, H15, H8) and control plots (DC13, DC5, DC3) were inoculated anaerobically into rhizosphere isolation media (RIM) (34) containing organosolv lignin as the sole carbon source instead of acetate. Organosolv is lignin derived from an ethanol-based separation process of lignin, hemicellulose, and cellulose from woody biomass, which produces a form of lignin closer to its original structural properties (35), ideal for determining if bacteria can break down and utilize raw material directly for pulping. We expected that our microbial community would be greatly reduced to members that could tolerate phenolics at the set concentration because lignin is known to have antimicrobial properties (36).

Over a total of 465 days, consortia were diluted 10^−3^ onto fresh RIM every four to 9 weeks. To confirm that consortia were viable, headspace gas composition was measured for CO_2_ production before and after each passage of the community with a Quantek 906 infrared gas analyzer (IRGA; Quantek Instruments, Grafton, MA, USA) (Fig. S1). DC13, DC5, and H16 were chosen for further analysis based on CO_2_ production levels. Direct cell counts using DAPI staining determined that the microbial biomass was 10^5^ cells/mL for all three consortia. To obtain isolates, DC13, DC5, and H16 were diluted to 1 to 5 cells/mL onto a 0.001% carbon mixture (wt/vol d-glucose, d-ribose, succinic acid, pyruvic acid, and glycerol; [37]) incubated in the dark at 25°C for 6 weeks anaerobically, then streaked onto R2A for colony picking. Purified isolated strains were maintained and routinely grown on the same medium and preserved at −80°C in 20% tryptic soy broth supplemented with 30% glycerol (vol/vol). To screen for lignin depolymerization capabilities, isolates were grown anaerobically on R2A plates containing lignin mimicking dyes, malachite green, and Congo red (18).

Colony and cell morphology of 159R were observed after a 24 h incubation at 30°C on R2A plates under oxic conditions. Growth at different temperatures (15 to 42°C) under oxic conditions was examined in liquid R2B (pH 7) to determine the optimal temperature for growth. In addition, a range of pH 4 to 10 at 30°C under oxic conditions in the R2B medium was used to determine the optimal pH for growth. Substrate utilization tests were performed with Biolog microplates under oxic conditions at 30°C for 24 h (Biolog GN2).

### 16S rRNA gene phylogeny.

To genotype strain 159R, the 16S rRNA (rRNA) gene was PCR amplified and sequenced using the primer pair 27F (5′-AGAGTTTGATCCTGGCTCAG-3′) and 1492R (5′-GGTTACCTTGTTACGACTT-3′). The raw sequence data were checked for accuracy, assembled, and edited using 4Peaks software version 1.8 (Nucleobytes, Aalsmeer, Netherlands). The 16S rRNA gene of strain 159R was then searched using the EZbiocloud service (http://ezbiocloud.net) and the GenBank database to identify its closest relative species. Tree estimation based on the 16S rRNA gene was completed with PASTA (version 1.8.5) (38). Strain 159R was aligned with organisms that were chosen based on previous literature on the *Sodalis*-clade, including *Sodalis*-like endosymbionts (8, 14). Sequences were taken from Ribosomal Database Project (RDP) (39).

### Genome sequencing.

To sequence and annotate the genome of strain 159R, cells were grown on R2A plates incubated for 3 days under oxic conditions at room temperature. Cells were scraped from the plate to extract genomic DNA (gDNA) using the Qiagen Genomic-tip protocol for bacteria.

The draft genome of strain 159R was generated at the Department of Energy Joint Genome Institute (DOE JGI). An unamplified library was generated using Pacific Biosciences standard template preparation protocol for creating 10 kb libraries (40). Briefly, 2.9 μg of unsheared gDNA was used to generate the library employing the Pacific Biosciences SMRTbell template preparation kit v1.0, where the fragments were treated with DNA damage repair so that they were blunt-ended, and 5′ phosphorylated. Pacific Biosciences hairpin adapters were then ligated to the fragments and exonuclease treated to remove failed ligation products and to create the SMRTbell templates for sequencing. Sequencing primer was then annealed to the SMRTbell templates and Version P6 sequencing polymerase was bound to them. The prepared SMRTbell template libraries were then sequenced on a Pacific Biosciences RSII sequencer using Version C4 chemistry and 2-h sequencing movie run times. 160,466 filtered subreads totaling 450,943,085 bp were generated. The raw reads were assembled using HGAP (smrt analysis/2.3.0 p5, HGAP 3) (41). Genome annotation was performed using the DOE JGI microbial genome annotation pipeline (MGAP v.4) (42). All metadata data curation and public repositories registration were managed by GOLD (43).

### Tree phylogeny.

The phylogeny of strain 159R was determined with three methods. The first method was to use multilocus sequence analysis (MLSA) via autoMLST, which produces a maximum likelihood tree. The program uses the top 100 gene homologs after searching with HMMER and other gene models (44). The second method also used the maximum likelihood algorithm via the KBase application Insert Genome into Species Tree 2.1.10 (45). This Kbase program combines genomes provided by the user with a set of closely related genomes selected from all public Kbase genomes. Based on alignment similarity to a select subset of 49 COG (clusters of orthologous groups) domains, the phylogenetic tree is then reconstructed using FastTree version 2.1.10 (46). The third method to determine the evolutionary relationship of *Sodalis* sp. strain 159R was with PhyloPhlan (47). This analysis uses 400 conserved proteins across the bacterial domain to produce a phylogeny using the maximum likelihood inference approach. Due to missing annotations, *Sodalis* sp. dw96 was not included in the PhyloPhlan tree. Visualization and editing of the trees were completed with iTol software version 3 (48).

### Genomic features and lignin metabolic potential.

Average nucleotide identity (ANI) was calculated using the MiSI Pairwise ANI tool from DOE JGI IMG/M (49, 50). The estimated genome-sequence-based digital DNA-DNA hybridization (dDDH) values were calculated with the Genome-to-Genome Calculator (GGC) software version 2.1, developed by DSMZ, using the formula 2 option as recommended (http://ggdc.dsmz.de/ggdc.php). The percentage of conserved proteins (POCP) between 159R and *Sodalis* members as well as close relatives B. albus and B. tofi was calculated as previously described (51). To determine the differences between strain 159R and *S.* ligni dw23, additional alignment analyses were completed with GSAlign (52) with visualization using d-Genies (via Minimap2 for the aligner) (53). GSAlign was run with default parameters and had strain 159R as the index and dw23 as the query.

Exploration of strain 159R’s functional potential and unique genes in relation to other *Sodalis* members was completed with JGI IMG/M’s Phylogenetic Profiler (49). In addition, using HMMER (v. 3.2.1), genes selected as markers for anaerobic aromatic metabolism were compared to the 159R genome (Table S1). Lignin-derived monomers with known anaerobic catabolic pathways include benzoyl-CoA, 3-hydroxy benzoyl-CoA, 3-methyl benzoyl-CoA, 4-methyl benzoyl-CoA, hydroxyhydroquinone, resorincol/α-resorcylate, and phloroglucinol (54, 55).

### Data availability.

Sodalis ligni str. 159R (=DSM 110549 =ATCC TSD-177) whole-genome shotgun project has been deposited in GenBank under accession no. SJOI00000000 (SRA accession no. SRX5645793 and SRX5645794). The GenBank Bioproject ID and Gold Project ID for this project are PRJNA524152 and Gp0312475, respectively. The 16S rRNA genes (totaling 7) have been deposited in GenBank under accession no. MT536223.1, MT536224.1, MT536225.1, MT536226.1, MT536227.1, MT536228.1, and MT536229.1.
