# Supplementary material for: Sodalis ligni Strain 159R Isolated from an Anaerobic Lignin-Degrading Consortium
Source: Microbiol Spectr. 2022 May 17;10(3):e02346-21. doi: 10.1128/spectrum.02346-21 (PMC9241852; doi:10.1128/spectrum.02346-21)
Supplement: SUPPLEMENTAL FILE 1 — Supplemental material. Download spectrum.02346-21-s001.pdf, PDF file, 0.5 MB [file spectrum.02346-21-s001.pdf]

A

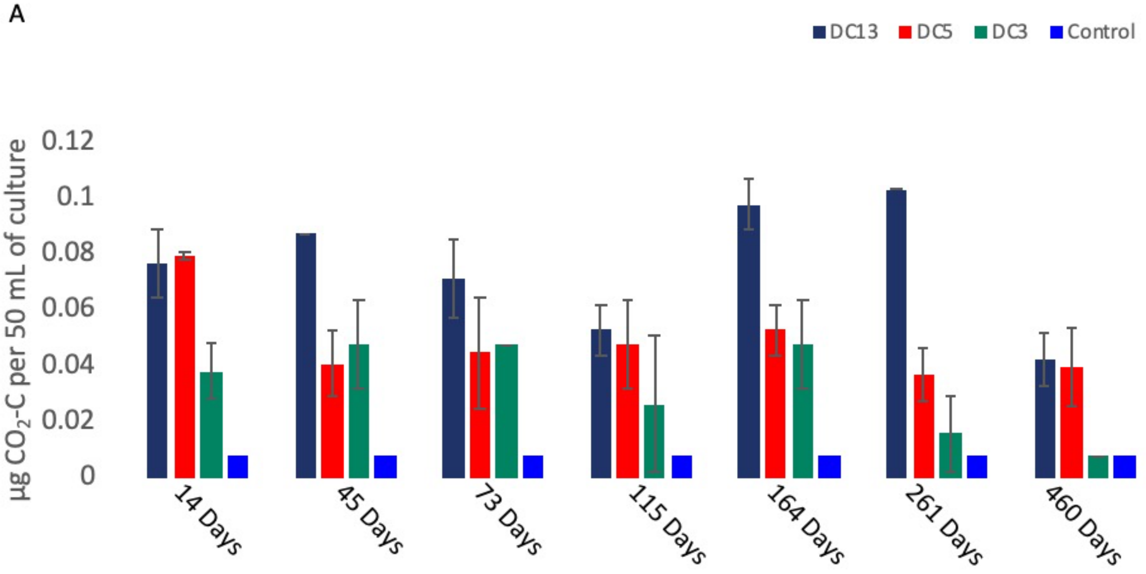

B

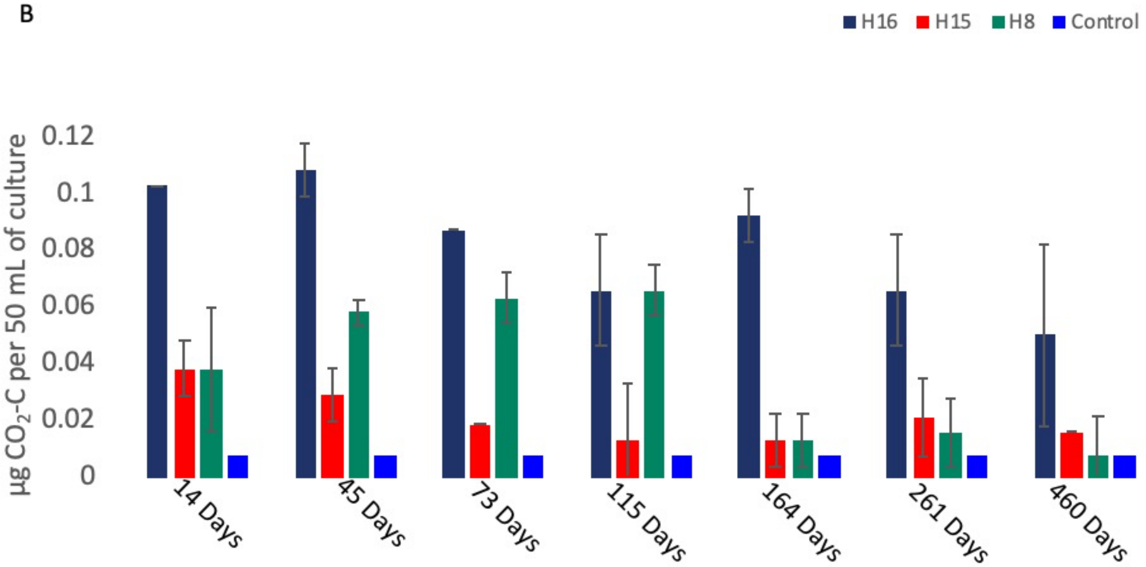

FIG S1 CO_2 production measurements (µg CO_2-C per 50 mL of culture) of microbial consortia inoculated originally from (**a**) control plots, with consortia labelled as DC13, DC5, and DC3, and (**b**) heated plots, with consortia labelled as H16, H15, and H8. Consortia were transferred at 1, 31, 59, 101, 150, 247, and 446 days, then allowed to grow for 2 weeks before respiration was measured to monitor growth. Abiotic controls are uninoculated consortia listed as "Control." Consortia DC13, D5 and H16 were selected for dilution to extinction culturing experiments.

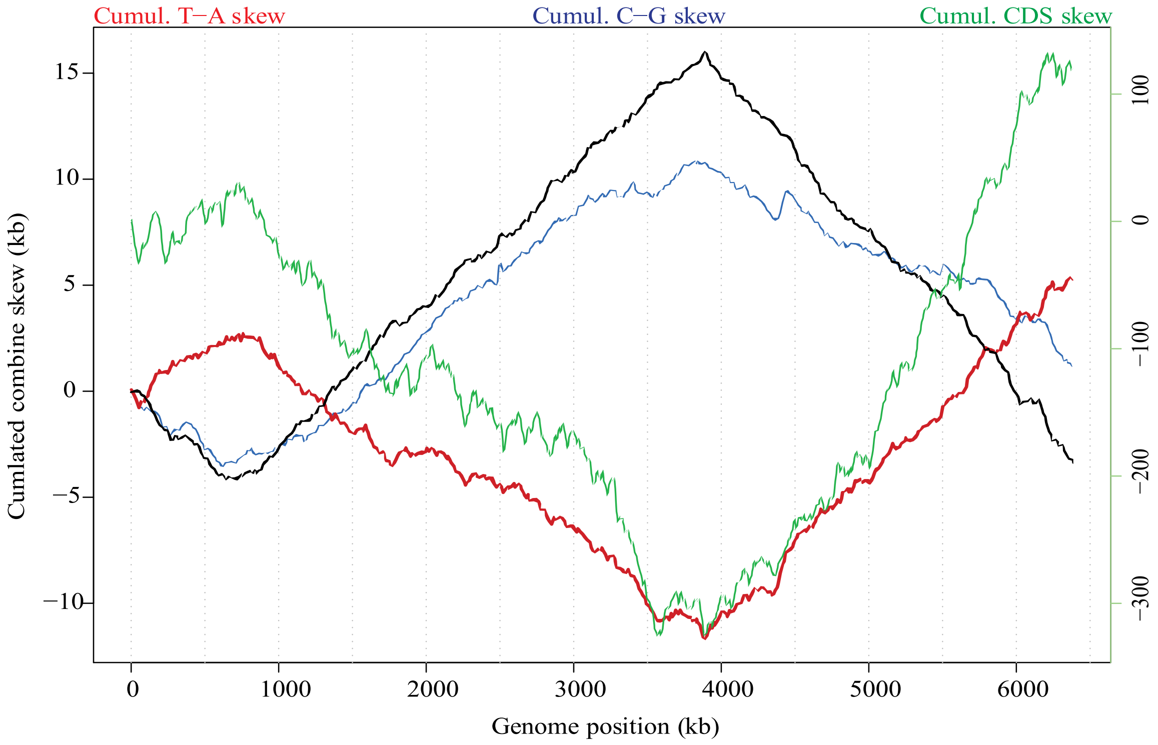

FIG S2 Cumulative GC(TA)-skew analysis of *Sodalis ligni* str. 159R using oriloc analysis. The cumulative combine skew is in black, the cumulative GC skew is in light blue, the cumulative TA skew is in red, and the cumulative coding sequences (CDS) skew is in green. The minimum and maximum of GC skew is used to predict the origin of replication at 3384 kb.

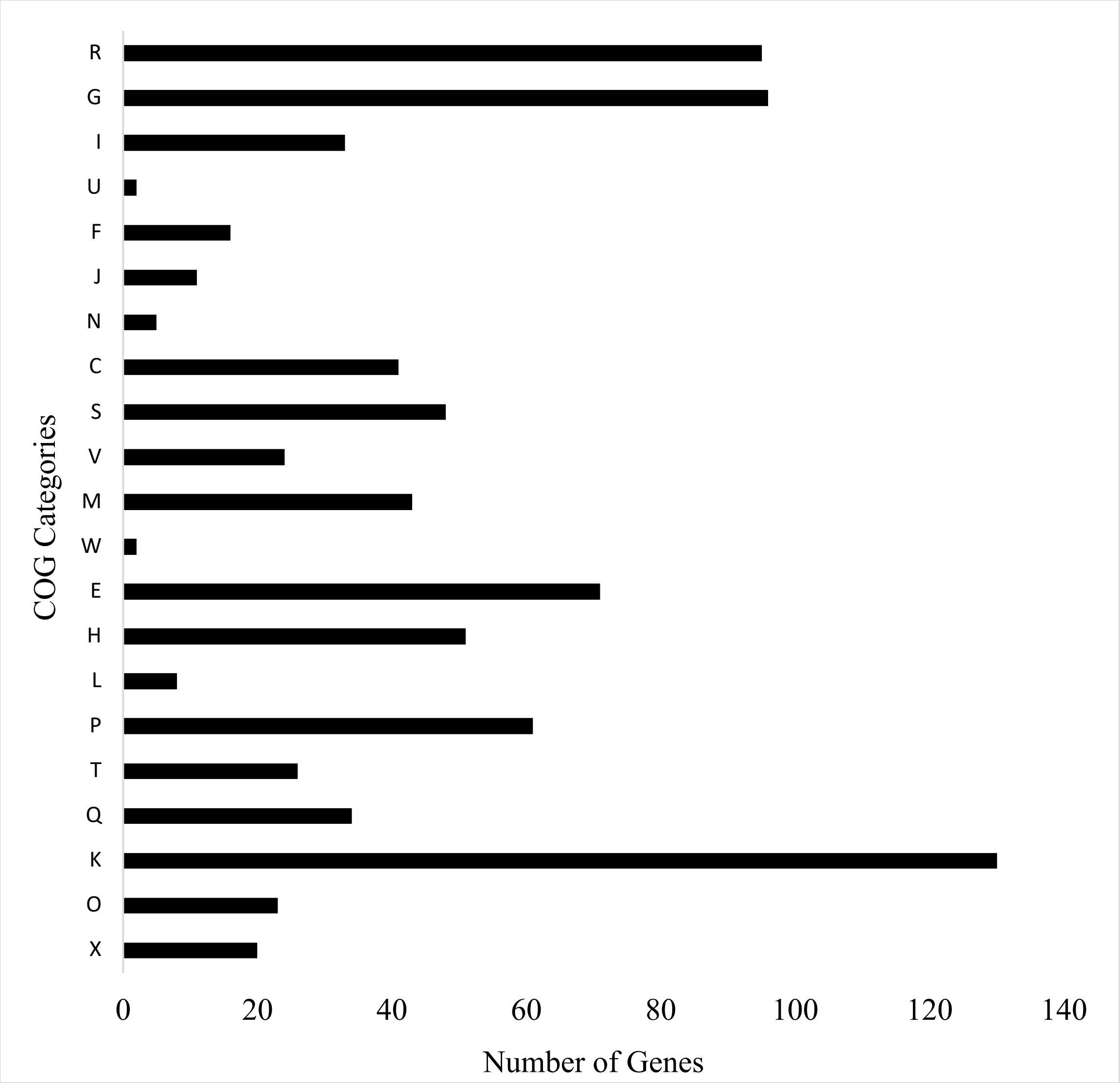

FIG S3 Unique gene abundance based on COG category for *Sodalis ligni* str. 159R compared to all organisms listed in Table 1. Abbreviations are as follows: (X) Mobilome: prophages, transposons; (O) Posttranslational modification, protein turnover, chaperones; (K) Transcription; (Q) Secondary metabolites biosynthesis, transport and catabolism; (T) Signal transduction mechanisms; (P) Inorganic ion transport and metabolism; (L) Replication recombination and repair; (H) Coenzyme transport and metabolism; (E) Amino acid transport and metabolism; (W) Extracellular structures; (M) Cell wall/membrane/envelope biogenesis; (V) Defense mechanisms; (S) Function unknown; (C) Energy production and conversion; (N) Cell motility; (J) Translation, ribosomal structure and biogenesis; (F) Nucleotide transport and metabolism; (U) Intracellular trafficking, secretion, and vesicular transport; (I) Lipid transport and metabolism; (G) Carbohydrate transport and metabolism; (R) General function prediction only.

TABLE S1 HMMER marker enzymes for anaerobic aromatic metabolism. Enzyme name is on the far let column followed by either the sequences used to build the profile Hidden Markov Model (HMM) with HMMER hmmbuild program or NCBI GenBank protein ID used for HMMER jackhmmer program. When applicable, subunits are listed separately.

**Benzoyl-CoA Pathway**	
Enoyl-CoA Hydratase	>SP|O87873|DCH_THAAR/7-257 Cyclohexa-1,5-dienecarbonyl-CoA hydratase [Thauera aromatica]<br>LKVWLERDGSLLRLRLARPKANIVDAAMIAAMRQALGEHLQAPALRAVLLDAE<br>GPHFSFGASVDEHMPDQCAQMLKSLHGLVREMLDSPVPILVALRGQCLGGGLE<br>VAAAGNLLFAAPDAKFGQPEIRLGVFAPAASCLLPPRVGQACAEDLLWSGRSID<br>GAEGHRIGLIDVLAEDPEAAALRWFDEHIARLSASSLRFAVRAARCDSVPRIKQK<br>LDTVEALYLEELMASHDAVEGLKAFLEKRSANWENR<br>>RF|YP_385104.1/6-256 enoyl-CoA hydratase/isomerase [Geobacter metallireducens GS-15]<br>LKVWLEKDGALLRLRLARPKANIVDAAMIAALQAALTEHLPSAKLRAVLLDAE<br>GPHFSFGASVEEHMPESCAAMLQSLHALVIQMLESPVPVLVAVRGQCLGGGLEV<br>VAAGNLIFAAPGAMLGQPEIKIGVFAPAASCLLPERIGKTASEDLLFSGRSITAEEG<br>FRIGLVTAVAEDPEQAAVAYFDEHLAGLSASSLRFAVRAARIGVLERTKTKIAAV<br>EKLYLEELMATHDAVEGLNAFLGKRPAAWQDR<br>>RF|YP_421505.1/9-259 Cyclohexa-1,5-dienecarbonyl-CoA hydratase [Magnetospirillum magneticum AMB-1]<br>LKVWKDREGKLLRLRLSRPKANIVDAEMIAALSAALGDAHEDSALRAVLIDHEG<br>PHFSFGASVAEHMPDQCAAMLASLHKLVIAMVDFPLPILVAVRGQCLGGGLEVA<br>LAGHMMFVSPDAKLGQPEIVLGVFAPAASCLLPERMPRVAAEDLLYSGRSIDGA<br>EAARLGIANAVVDDPENAALAWFDNGPAKHSAASLRFAVKAARLGMNERVKA<br>KIAEVEALYLNGLMATHDAVEGLNAFLEKRPALWEDR
Hydroxyacyl-CoA Dehydrogenases	>OMNI|NTL01AE3009/8-353<br>TWQMTEPGK-<br>LQKTRVPMPELGSGDVVVKIAGCGVCHTDLSYFYMGVPTVQKPPLSLGHEISGTI<br>I---<br>GGEASMIGKEVIVPAVIPCGECELCKTGRGNRCLAQKMPGNSMGIYGGYSSHIV<br>AQSKYLCVVEN----<br>RGDTPLEHLAVVADAVTTPYQAAVRADLKKDDLVIVVGAAGGVGSFMVQTAK<br>GMGAKAVIGIDINEEKLEMMKGFGADFIINPKDK-SAKEVKELFKGFCKE<br>RGLPSNYGWKIFEVTGSKPGGQELALSLLSFTGKLVIVGYGTAETNYMLSKLMAF<br>DAEIIGTWGCPPDRYAAVRDMCLDGRIQLGPFVETRPMSQIEHVFDEAHHGKLK<br>RRVILTP<br>>gi|19571180/20-368 6-hydroxycylohex-1-en-1-carbonyl-CoA dehydrogenase [Thauera aromatica]<br>RWMMTSPGAPMVRAEFEIGELSADQVVVAVAGCGVCHTDLGYYYDSVRTNHA<br>LPLALGHEISGRVVQAGANAAQWLGRAVIVPAVMPCGTCELCTSGHGTICRDQV<br>MPGNDIQ--GGFASHVVVPARGLCPVDEARLAAAGLQLADVSVVADAVTTPYQA<br>VLQAGVEPGDVAVVIGV-GGVGGYAVQIANAFGA-<br>SVVAIDVDPAKLEMMSKHGAALTLNAREI-SGRDLKKAIEAHAKANGLRLT-<br>RWKIFECSGTGAGQTSAYGLLTHGATLAVVGFTMDKVEVRLSNLMAFHARALG<br>NWGCLPEYYPAALDLVLDKKIDLASFIERHPLDQIGEVFAAAHAHKLTRRAILTP<br>>OMNI|NTL06MM2144/25-374<br>RWMMTGVGQPMVKEAMEIAAPGAGEVLVEVAGCGVCHTDLDYYYNGVRTNH<br>ALPLALGHEISGRVIQAGAGAESWVGKAVIISAVIPCGQCDLCKRGKGTICRSQK<br>MPGNDLQ--<br>GGFATHITVPANGLCAVDEARLKAAGLELSEVSVVADALTTPYQAAVQAGIGQG<br>DLVIVIGC-GGVGGYSVQVASAMGA-<br>TVVALDIDPVKLEAVKAAGAKLTLNPKDFPSTREIKKEIGAFAKAQGLRST-

	EWIIMECSGSVPGQQSAFDLMVHGCTICVVGYTMNKAEFRLSNLMAFHARALG<br>NWGCPPDLYPGALDLVLSGKINVKNFVERRPLDSINDTFAAVHDHKLSRRAVLC<br>P
Oxoacyl-CoA hydrolase	>OMNI|NTL01AE3010/12-371<br>IKDHALMGEEHFGTEAPSVL-<br>FEKRPVTDPQGNVVPGLYAAWIILNNPKQYNSYTTEMVKAIIAGFQRASSDRTIVA<br>AVFTAVGDKAFCTGGNTAEYASYYAQRPNEYGEYMDLFNAMVDGILNCKKPTIC<br>RVNGMRVGGGQEIGMATDLTITSDMAIFGQAGPKHGSAPDGGSTDFLPWMLNME<br>DAMYNCISCEPWSAYKMKSKNLITKVVPVLKKDGEWVRNPLVRTDAYVDD-<br>GELV<br>YGEPVAADKAKAAKELIAQCTTDFAKLDEAVDALVWKFANLFPQCLIKSIDGIRG<br>KKKFFWDQMKLANRHWLAANMNHEAYLGFTAFNN-<br>KKATGKDVIDFIKFRQLVAEGHAFDDAFAEQVL<br>>OMNI|NTL01GM2088/16-376<br>LNDHNLIDRE-<br>VESLCDGMVKYEKRPAKRHDGSVAEGIYNAWIILDNPKQYNSYTTDMVKAIILAF<br>RRASVDRSVNAVVF<br>TGVGDKAFCTGGNTKEYAEYYAGNPQEYRQYMRLFNDMVSAILGCDKAVISRV<br>NGMRIGGGQEIGMACDFSIAQDLANFGQAGPKHGSAAIGGATDFLPLMVGCEQA<br>MVSGTLCEPFSAHKAARLGIICDVVPALKVGGKFVANPTVVTDRYLDEYGRVVH<br>GEFKAGAAFKEGQGQIKEGEIDLSLLDEKVESLCTKLLETFPECMTKSLEELRKPK<br>LHAWNLNKENSRAWLALNMMNEA<br>RTGFRAFNEGTKETGRE-IDFVKLRQGLAKGTPWTEELIESLM<br>>OMNI|NTL06MM2143/17-372<br>LNDHNLV----PTTVVPGVL-<br>YEKRPAKRADGTVAEGLYNAWITLDNQKQYNSYTTDMVKGVIMAFRDASNARD<br>VSSVVF<br>TGAGDKAFCTGGNTKEYAEYYAGNPQEYRQYMRLFNDMVSAILGCDKPVICRV<br>NGMRIGGGQEIGMAADFSVAQDLAKFGQAGPKHGSAPIGGATDFLPVMIGCEQA<br>MVSGSLCEPWSAHKAYRTGIIMDLVPALKVDGKFVANPLVITDRYLDEFGKIVHG<br>ESKTGAELAAGKELLKKGTIDLSLLDAKVEEICAKILHTFPDCFTKTIQELRKPKLN<br>AWNANKENSRDWLGLNMMTEARTGFRAFNEGPKE-DRE-<br>IDFVALRQALAKGAPWTPELIESLI<br>>gi|3724166/17-373<br>LVDHNLV----PETVCPGVL-<br>YEKRPARNLKGEVVPGLYNVWISLDNPKQYNSYTTDMVKGLILAFRAASCARDV<br>ASVVF<br>TAVGDKAFCTGGNTKEYAEYYAGNPQEYRQYMRLFNDMVSAILGCDKPVICRV<br>NGMRIGGGQEIGMAADFTVAQDLANFGQAGPKHGSAAIGGATDFLPLMIGCEQA<br>MVSGTLCEPFSAHKANRLGICMQIVPALKVDGKFIANPLVVTDRYLDEFGRIIHGE<br>FKTGDELAAGKELMKRGEIDLSLLDEAVEKLCAKLISTFPECLTKSFEELRKPKLD<br>AWNRNKENSRAWLALNMMNEARTGFRAFNEGNKETGRE-<br>IEFTDLRQALAKGMPWTPELIESLM
**3-Hydroxybenzoyl-CoA Pathway**	
Enoyl-CoA Hydratase	>WP_050418522.1 enoyl-CoA hydratase/isomerase family protein [Azoarcus sp. CIB]<br>MISLRIEDS------------VATVTLCRAPV-NAINEEWIAAFDRILAELEHTPRVNVL<br>WIRSAERVFCAGADL-<br>DVIGSLFATEAGRVQMIAITRRMQQLYARLERLPQVTVAEIGGA<br>AMGGGFELALACDLRVVADSAKVGLPEARLGLLPAA-<br>GGTQRMTRICGEAVARRLILGAE<br>VVGGVDAVKLGCAHWVAPAAELEEFTRGVVTRIAALPALALSECKRCITVAVE<br>GD-EDGY<br>QVELAGSAALLADGETQQRVRAFLNR--------<br>>WP_011236223.1 enoyl-CoA hydratase/isomerase family protein [Aromatoleum aromaticum]<br>MISLTIEAS-----------VATVTLCRSPV-NAINEEWIEQLDRILAEIERTPRVNVL

	WIRSGERVFCAGADL-<br>ELIRSLFDSETGRRQMIAMTRRMQEVYARLERLPQVSVVEIGGA<br>AMGGGFELALACDLRVVADSARIGLPEARLGLLPAA-<br>GGTQRMTRICGEAVARRLILGAE<br>VIGGAEAVALGCAHWVAPAAELESVARAVVERIAALPGTALAECKRCIDVAVA<br>AE-ENGF<br>EVELSGSAALLADAETQRRVQRFLDKQRQ-----<br>>CAC28159.1 putative hydrolase [Thauera aromatica]<br>MSVVLVEQPTPD---------<br>VAVVRLNRPDARNALNQEVRSALAEHFDRLGQAAEVRCI<br>VLTGGERCFAAAPDIRAM------ADAG--<br>AIEIMLRQTQRLWQAIAACPKPVIAAVNGY<br>AWGGGCELAMHADIIIAGEGASFCQPEVKVGIMPGA-<br>GGTQRLTRAVGKFQAMKMVLTGL<br>PVSARERLAMGLASEVVADDAVQARALELARHIATLPPLAIAQIKEVLLAGQDA<br>SLDTAL<br>MLERKAFQLLFASADQKEGMRAFLEKRPPVFRGG<br>>CAC28155.1 unnamed protein product [Thauera aromatica]<br>MYKLKAADWHPEHFKLEVANRVATITLNRPDRKNPLTFESYAELRDTFHKFQY<br>VDDVRSI<br>VITGAGGNFCSGGDVHDIIGPLTKMDMN--<br>GLLTFTRMTGNLVKEMRTCPQPIISAIDGI<br>CAGAGAIVSMASDMRYATPDAKTAFLFVRVGLAGCDMGACAILPRIIGHGRASE<br>LLYTGR<br>VMSAQEGQAWGYFNDLVAPDQVLAKAQEMALSLANGPAFAHAMTKKCLHQE<br>WDMSIEQAL<br>ETEAEAQAICMQTQDFTRAYNAFVAKQKPVFEGN<br>>WP_050418021.1 enoyl-CoA hydratase family protein [Azoarcus sp. CIB]<br>MYKLKAAEWRPEHFKLEVADRVATITLNRPERKNPLTFESYAELRDTFIKLQYA<br>EDVRAV<br>VMTGAGGNFCSGGDVHDIIGPLTKMDMT--<br>GLLAFTRMTGNLVKEMRNCPQPIISAVDGV<br>CAGAGAIITMASDLRYATPEAKTAFLFVRVGLAGCDMGACSILPRIIGQGRASEL<br>LYTGR<br>SMSAEEGRAWGYFNDVVPAEKVLAKAQEMALSLANGPAFAHSVTKKCLHQEW<br>NQTIEQAL<br>ETEAEAQAICMQTEDFTRAYNAFVNKQVPKFEGN<br>>WP_011236224.1 enoyl-CoA hydratase family protein [Aromatoleum aromaticum]<br>MYKLKAAEWRPEHFKLEVADRVATITLNRPERKNPLTFESYAELRDTFHKLQYV<br>DDVRTV<br>VITGAGGNFCSGGDVHDIIGPLTKMDMN--<br>GLLTFTRMTGNLVKEMRNCPQPIISAVDGI<br>CAGAGAIVSMASDLRYATPEAKTAFLFVRVGLAGCDMGACSILPRIIGHGRASEL<br>LYTGR<br>SMSAEEGRAWGYFNDIVPAEKVLGRAQEMALSLANGPAFAHSMTKKCLHQEW<br>NQTIEQAL<br>ETEAEAQAICMQTQDFTRAYNAFVNKQVPKFEGN
Hydroxyacyl-CoA Dehydrogenases	>WP_050418028.1 SDR family oxidoreductase [Azoarcus sp. CIB]<br>MTADSGRALAGKHVVITGGGRGIGAAIAAALSAQGARLTLMGRNRGQLEER--<br>AAVLRTL<br>GGESCEVHCEAVDVADEASVVSAFAAAAKRLGPVAVLVNNAGQAGSAPFLRTE<br>SALWQQM<br>LAVNLTGTYLATRAALPDMLAAG-<br>WGRIINVASTAGEKGYPYVTAYCAAKHGVIGLTRSL<br>ALELAHKHVTVNAVCPGYTDTDIVRDAVTNIREKTGRSEAEALAELAKHNPQGR<br>LVRPEE

	VANAVLWLCLPGSDAITGQAISVSGGEVM--<br>>CAC28156.1 putative alcohol dehydrogenase [Thauera aromatica]<br>--MTHSRALSGKHAVITGGGRGIGAAIAHSLAEQGAAVTLMGRTLPRLEQQ--<br>AEELRAF<br>SQ----<br>VHCEAVDVAQADSVAAAFAAAQARLGPVDILVNNAGQALSAPFVKTDPALWQ<br>QM<br>LDVNLTGVFLGTRAVLPGMLAAG-<br>WGRVINITSTAGQKGYPYVSAYCAAKHGVIGLTRAL<br>ALETARKNVTVNAVCPGYTDTDIVRDSVSNIQTKTGRSEAEALAELTRFNPQGRL<br>VRPQE<br>VANAVLWLCLPGSEAITGQSISVAGGEMM--<br>>WP_041646819.1 SDR family oxidoreductase [Aromatoleum aromaticum]<br>-----MRELSGKHAVVTGGGRGIGAAIAQRLAEQGACVTLMGRRREPLEER--<br>ADALRAL<br>IGVHCDMHCEAVDVADPASVAAAFDAAARRFGPVSILVNNAGQASSAPFVKTD<br>LALWQRM<br>LDVNLTGTYLGTKAVLSGMLAAG-<br>WGRIVNVASTAGQKGYPYVSAYCAAKHGVIGMTRAL<br>ALELAQKNITVNAVCPGYTDTDIVREAITNIRAKTGRSEAEAQGELAKHNPQGRL<br>VRPDE<br>VANAVLWLCLPGAEAITGQAISVSGGEVM--<br>>CAC28154.1 putative alcohol dehydrogenase [Thauera aromatica]<br>------MRLEGKTAVVTGGASGIGRATAETLAAAGAHVVI-----<br>GDLDQEKGAAVAAAI<br>RESGRKADYFPLDVTSLDSVGVFAKAVEENGLEVDIVVNVAGWGKIQPFMENSP<br>DFWRKV<br>IDLNLLGPVAVTHAFLGGMIARGRGGKVITVASDAGRVGSTGETVYSGAKGGAI<br>AFGKAL<br>AREMARYKINVNSVCPGPTDTPLLAAVPEKHQE-----------<br>AFVKATPMRRLGKPSE<br>IADAVLFFASSDSDFITGQVLSVSGGMTMVG<br>>WP_011236225.1 SDR family oxidoreductase [Aromatoleum aromaticum]<br>------MRLDGKTAVVTGGASGIGLATAETLARAGAYVLI-----<br>GDIDEQKGAAVAGAL<br>CEQQLGVDFIRLDVTDLDSIAAFKDEAYRRRPQIDIVANVAGWGKIQPFMENTPD<br>FWRKV<br>IDLNLLGPVAVSHAFLPQMIERG-<br>AGKIVTVASDAGRVGSLGETVYSGAKGGAIAFTKSL<br>AREVARYNINVNCVCPGPTDTPLLQAVPEKHRE-----------<br>AFVKATPMRRLAKPSE<br>LADAVLFFASDRASFITGQVISVSGGLTLAG<br>>WP_050418022.1 SDR family oxidoreductase [Azoarcus sp. CIB]<br>------MNLQGKTAVVTGGASGIGYATAETLARAGAKVVI-----<br>GDIDAAKGAAAAGML<br>AEQHLDVDFVRLDVTDIDSIHAFRDETYRRHPQVDIVANVAGWGKIQPFMENTP<br>DFWRKV<br>IDLNLLGPVAVSHAFLQQMIERG-<br>SGKIVTVSSDAGRVGSLGETVYSGAKGGAIAFTKSL<br>AREVARYNINVNCVCPGPTDTPLLQAVPEKHRE-----------<br>AFVKATPMRRLAKPSE<br>LADAVLFFASDRASFITGQVISVSGGLTLAG
Oxoacyl-CoA hydrolase	>CAC28157.1 putative acyl-CoA dehydrogenase [Thauera aromatica]<br>MSEKSYLEWPFFEDRHRKLEAELDSWATNNISEHH-<br>GELDSACRELVAKLGAAGWLRYCV

	GGTSYGGEHETIDTRSICLLRETLARHSGLADFAFGMQGLGSGAITLHGSDAQKR<br>EYLPR<br>VASGQALAAFALSEPGSGSDVAAMACSARLDGEYYVLDGEKSWISNGGIADFY<br>VVFARTG<br>EAPGARGLSAFIVDADTPGLEIAERIEVIAPHPLARLRFTDCRVHKSAMLGTPGLG<br>FKVA<br>MQTLDIFRTSVAAAALGFSRRALDEALRRATTREMFQQKLADFQITQVKLAQMA<br>TSVDIS<br>ALLTYRAAWRRDQGHKVTREAAMAKMTATESAQQVIDSAVQIWGGCGVVSNH<br>PVELLYRE<br>IRALRIYEGATEVQQLIIARQTLTAYEDS---<br>>WP_050418027.1 acyl-CoA dehydrogenase [Azoarcus sp. CIB]<br>MSDRSYLEWPFFEERHRGMQVELEAWAAAHIDGHPHGDLDDACRELVRKLGA<br>DGWLRYMV<br>GGTAYGGRHDTIDTRAVCLLRETLARHSGLADFALGMQGLGSGAITLHGTDAQ<br>KRKYLSE<br>VAAGRAIPAFALSEPDSGSDVAAMACSARRDGNDYVLDGEKTWISNGGIADFYV<br>VFARTG<br>EAPGARGLSAFIVEANLPGFEIAERIDVIAPHPLARLRFTGCRVPAANLLGAPGQG<br>FKVA<br>MQTLDIFRTSVAAAALGFARRALDEGLRRATTRDMFGKKLADFQITQAKLAQM<br>ATHVDTA<br>ALLTYRAAWMRDQGKNITGAAAMAKMTSTETAQQVIDAAVQLWGGCGVVSE<br>HPVERLYRE<br>IRALRIYEGATEVQQLIIARQTLSAWEQEQAV<br>>WP_011236231.1 acyl-CoA dehydrogenase [Aromatoleum aromaticum]<br>MSDQTYLEWPFFDEPHRQLQIELEAWASANVTEHHGSDLDTACRELVAKFGAA<br>GWLRYVV<br>GGTAYGGCHDVIDTRAVCLLRETLGRHSGLADFAFGMQGLGSGAITLHGTDAQ<br>KRDYLPR<br>VASGRAIAAFALSEPGSGSDVAAMACSARQDGDEYVIDGEKTWISNGGIADFYV<br>VFARTG<br>EAAGSRGLSAFIVDADRPGLEIAERIDVIAPHPLARLRFRECRVPKSCLLGVPGQG<br>FKVA<br>MQTLDIFRTSVAAAALGFARRALDEALKRATTRDMFGQKLADFQITQAKLAQM<br>ATAVDTS<br>ALLTYRAAWLRDQGQTITGAAAMAKMTSTETAQQVIDAAVQMWGGCGVVSD<br>HPVERLYRE<br>IRSLRIYEGATEVQQLIIARQTLSAYERQQEH
**3-Methylbenzoyl-CoA Pathway**	
Enoyl-CoA Hydratase	CCH23021.1
Hydroxyacyl-CoA Dehydrogenases	CCH23023.1
Oxoacyl-CoA hydrolase	CCH23022.1
**4-Methylbenzoyl-CoA Pathway**	
Enoyl-CoA Hydratase	AIW63094.1
Hydroxyacyl-CoA Dehydrogenases	AIW63095.1

Oxoacyl-CoA hydrolase	AIW63096.1
**Resorcinol Pathway**	
3,5-dihydroxybenzoate hydroxylase large subunit (DbhL)	AIO06084.1
3,5-dihydroxybenzoate hydroxylase small subunit (DbhS)	AIO06085.1
Resorcinol hydroxylase large subunit (RehL)	ABK58620.1
Resorcinol hydroxylase large subunit (RehS)	ABK58619.1
**Hydroxyhydroquinone Pathway**	
Benzoquinone Dehydrogenase BqdL	>AIO06095.1 benzoquinone dehydrogenase alpha subunit [Thauera aromatica]<br>MPKTIDLHYHAPWQEVVATADDWDHLGSATVLRMLHHLHLVRAFEETVLELD<br>GEGLVHGP<br>AHSSIGQDGGAVGAVSLLRSSDLITGSHRGHHQFLAKCLAHLDRGEADPRRTPLS<br>EGVRT<br>MLYRALAEILGLADGYCRGRGGSMHLRWAEAGALGTNAIVGGGVPLATGAAW<br>ACKRRGAG<br>DVAFTFLGDGAVNIGAVPESMNLAALWSLPVCFFIENNGYAVSTKLSEETRETRL<br>SSRGG<br>AYGIPALRVDGMDPVAVRVATQMALDAMRAGQGPYIIEAEVYRYFHHGGGLPG<br>SAFGYRS<br>KDEEAAWRARDPLACLARGMIERDWLSADEDATLRAGARACMVEIAARLTEK<br>DGSKRRIV<br>PALWPQATFRDEGVRGDLAELAGVRCEELETASGKVGEVKFISAVAGVMARRM<br>ESDERIF<br>CLGEDIHKLNGGTNGATRGLAARFPDRIVPTPIAEQGFVGLAGGVAMEGHYRPV<br>VELMYA<br>DFALVAADPLFNQIGKARHMFGGDMAVPLVLRSKCAIGTGYGSQHSMDPAGLY<br>AMWPGWR<br>IVAPSTPFDYVGLMNSALQCDDPVLVIEHVGLYNTTAPGPLEDFDYYIPLGKAKV<br>VRPGT<br>ALTVLTYLAMTPLAVKVADELGVDAEVIDLRSLDRAGIDWETIGDSVRKTNNVV<br>VLEQGS<br>QTASYGAMLADEVQRRLFDHLDQPVKRIHGGEAAPNVSKVLERAAFVGAEEVR<br>AGFIEVL<br>ADAGRPLAQTAPALG-----<br>>ABK58621.1 dehydrogenase [Azoarcus anaerobius]<br>MPRITNLDYAEPWIELASTPQDWKKLGKTELLRVLYYHHLVRAFEEAVLNLEKL<br>GLVHGP<br>AHSSIGQEGGAVGSVMLLNSSDMITGAHRGHHQFLVKGMQHIDSPSYDPRAAPL<br>PEEVQT

	FLYRTLAEILGLSDGFCKGRGGSMHLRWVEAGAMGTNAIVGGGVPIANGLAWA<br>QKRRNKG<br>EVTFTFFGDGGMNIGAVPESMNLAALWNLPICFFIENNGYAVSTTLEEETRETRL<br>SSRGG<br>AYAIPAWRVDGMDPVAVRLASEAAIERMRAGKGPTIIEAVLYRYFHHGGSVAGS<br>AFGYRK<br>KDEESSWIAKDPLDRTVREMINLQWLTADENTAIRRHCESAMQGIVERLVEGEG<br>SKRRIR<br>AELWPKPEFRDQGLRGDLSEFKDARFEELETASGPVGDVKFVDAVARVMGRRM<br>ETDERVF<br>CMGEDIHRLKGGTNGATKGLAERFPDRIIPAPIAEQGFVGLAGGVAQDGQYRPV<br>VELMYS<br>DFALVAADQLFNQIGKARHMFGGDSAVPLVLRTKCAIGTGYGSQHSMDPAGMY<br>AMWPGWR<br>IVAPSTPFDYVGLMNSALKCEDPVLVIEHTDLYNTTDQGPLEDLDYCIELGKAKV<br>VRKGS<br>AFTVLTYLAMTPLALKVADEMGLDVEIIDLRSLDRAGIDWATIGESIRKTNNVVV<br>LEQGP<br>LTVSYGAMLTDEIQRRFFDYLDQPVQRIHGGESSPSVSKVLERAAFVGAEEIRAG<br>FTRMM<br>ADMGQPLPATPSPAGNSITA
Benzoquinone Dehydrogenase BqdS	>AIO06106.1 benzoquinone dehydrogenase small subunit [Thauera aromatica]<br>MPVEILMPSTGASMSEGNILRWLKQEGEAVERGEALLEIETDKAVVEAVTPARGI<br>LGKIL<br>AAGGSEGVKVDSVVGLIAVDGEDPVALAGAVLAGATPAGSAPAGAATVATA----<br>-----<br>AGEASPAEVQRRIPASPLARRLARETGVDLAAVRGRGPHGRVLRADVESVARQA<br>AAAAAP<br>GGAAPLLAATVAAAGTAVPSAAGAAFEDIPHSAMRRVIAQRLGEAKRTVPHFYL<br>SLDCAV<br>DALLALRAQINAQLDAQVGAQVGAQVGAHPDGGKLSVNDFIVKAVALALRRVP<br>GCNAAWT<br>EAAVRRFAEVDIAVAVATPGGLITPIVRHADDKSLGSLSAEIRALAGRAREGRLK<br>PEEYQ<br>GGGFTLSNLGMYGIREFAAIINPPQACILAVGACEQRPVVRDGSLAVATLMSCTL<br>SVDHR<br>VVDGAQAAEFLAEFRRLIENPLAILV<br>>ABK58622.1 dihydrolipoamide acetyltransferase [Azoarcus anaerobius]<br>------<br>MPSVSTSMTEGTLARWLKKDGETVAKGEVIAEIETDKAILEVEAEAEGIFKAFV<br>ADGAT--<br>VKVGEPMGALLAPGETLGGTISAAQSAAAPTAAAVGGETAVAVAVAAPAAAPS<br>TGHAPAAHDGTRIFASPLARSLALLHGLDLVNISGSGPQGRIVKRDIEA-<br>AMSAQRPASG<br>AVAAPVAEAPVKAPQPAAPQAAGAGYELIPHSSMRRVIAQRLSESKQQVPHFYL<br>TVDCRL<br>DKLLALRQQVN---------------GSLPD-VKVSVNDFIVKAVAAAMKRVPATNASWS<br>DEGVRRYRDIDISVAVATPNGLITPVVRQADAKSVGTISAEVKDLAERARQGKL<br>KPDEYQ<br>GGGFTISNLGMYGVRDFAAIINPPQACILAVGTAEKRPVIEDGAIVPATVMTCTLS<br>VDHR<br>VVDGAVGAEFLAAFKALLETPLGLLV
Benzoquinone Dehydrogenase BqdM	>ABK58623.1 putative dehydrogenase E3 component [Azoarcus anaerobius]<br>-<br>MAQEKFDLTVIGGGPGGYVAAIRAAQLGLRTALIEREHLGGICLNWGCIPTKALL<br>RSAE

	IFDHFKHAGDFGLEVQGASFDLQKIVARSRGVAAQLNAGVKHLLKKNKVQVFE GSGRLAG SGTIRLEQKDG- VSEIQSTHIILATGARARAMAPVEPDGRLVWSYKEAMTPERMPKSLLI VGSGAIGIEFASFYRSLGAEVTVVEVRDRVLPVEDAEVSAFAHKAFERQGMKLL TSSSVV SLQKQADSVIAVIDTKGTTTEIRADRVIAAVGIVGNVENLGLEGTGVQVENTHIV TDAWC QTGEPGVYAIGDVAGAPWLAHKASHEGILCVERIAGVDGIHPLDKTRIPGCTYSR PQIAS IGLTEAQAKERGYELKVGRFPFMGNGKAIALGEPEGFIKTVFDAKTGELLGAHM VGAEVT ELIQGFSIGKTLETTEAELMHTVFPHPTLSEMLHEATLAAYGRAIHT<br>>AIO06092.1 dihydrolipoamide dehydrogenase family protein [Thauera aromatica]<br>MTDNNSYDLIVVGAGPGGYVAAIRAAQLGMKTAVVEREHLGGICLNWGCIPTK ALLRSAE VGRLARHAAEYGVSVPEPKFDLERIVQRSRAIAAQLNGGIRHLLNKNKVSVIEGE ARLAG AGRVAVTRGGADAGTYAAPHLILATGARARQLPGLEDDGRLVWTYRKAMTPD VLPKSLLI VGSGAIGIEFASFYHALGSQVTVVEVMDRILPVEDEDISALARKAFEDQGMRILT GAKAS IARKSAECVTVRIEAGGAAEELTVDRVIVAVGISPNTENLGLEHTRVRLERGHIVT DPWC RTDEPGLYAIGDVTRPPWLAHKASHEAMICVEAIAGLADVHPLELRNIPGCTYSH PQIAS VGLTERKAREQGHEVRVGRFPFVGNGKAIALGEPEGLVKTVFDARSGELLGAH MIGAEVT ELIQGYTLARTLEATEAELIATVFPHPTLSETMHEAVLAAYGRAIHI
HHQ dehydrogenase large subunit (BtdhL)	ABK58630.1
HHQ dehydrogenase small subunit (BtdhS)	ABK58631.1
**Phloroglucinol Pathway**	
Phloroglucinol Reductase	>WP_014184752.1 SDR family oxidoreductase [Desulfosporosinus orientis]<br>MVDIQ-- FVNNLFDVKDKVALITGATGALGKAISFGYGLAGMKIFVTGRSGEKCKALCDE LEAQGIECGYSIGDPAVEADVIKVVEDAVQKFGEINVLLTAAGYNHPQPIVDQDL AEWKK IMDSDVQGTWLFCKYAGQQMIERGKGGKVILVSSARSKMGMAGYTGYCTAKA GIDLMAQS LACEWTAKYKINVNTINPTVFRSDLTEWMFDPESPVYANFLKRLPVGRLGEPEDF IGPCI FLASNASDFMTGANVATEGGYWAN<br>>WP_021630531.1 SDR family oxidoreductase [Clostridium sp. ATCC BAA-442]<br>MVNVKKEFVDNMFSVKGKVALVTGATGALGCVLSKAYGYAGAKVFMTGRNE KKLQALEAE FKAEGIDCAYGVADPADEAQVDAMITACVAQYGEVNILAVTHGFNKPQNILEQS VADWQY IMDADCKSVYVVCKYVAQQMVDQGKGGKIVVVTSQRSKRGMAGYTGYCTSK GGADLMVSS

	MACDLSAKYGINVNSICPTVFRSDLTEWMFDPESAVYQNFLKREPIGRLAEPEDF<br>VGYAL<br>FLSSDASNYITGANCDCSGGYLTC<br>>WP_027868985.1 SDR family oxidoreductase [Eubacterium sp. AB3007]<br>MVNVEKSFVNNMFSVEGKVALVTGATGALGCVLSKAYGYAGAKVFMTGRNAE<br>KLQKLQDE<br>FEAEGIDCAYFVADPQKEEDVKALIAACVEKYGEVNILAICHGYNKPANILDQSV<br>EDWQF<br>IMDADCKSVYIVCKYVAEQMVEQGKGGKMVVVTSQRSKRGMAGYTGYCTSKG<br>GADLMVSS<br>MACDLTAKYGINVNSICPTVFRSELTEWMFDPDSEVYKNFLKREPIGRLAEPYDF<br>VGFAL<br>FLSSEASDFMTGGNYDCSGGYLTC

TABLE S2 Carbon substrate utilization of strains *Sodalis ligni* str. 159R (159R), *S. praecaptivus* HS (HS), *S. glossinidius* (Sgl), and *Biostraticola tofi* (Bto). Data not available: ND.

	159R	HS	Sgl	Bto
α-D-Glucose-1 Phosphate	+	ND	ND	+
α-D-Glucose	+	+	+	+
α-D-Lactose	+	+	–	+
Cellobiose	–	+	ND	+
D-Glucose-6-Phosphate	+	ND	ND	+
D-Fructose	+	+	–	+
D-Galactonic Acid Lactone	+	ND	ND	+
D-Galactose	+	+	–	+
D-Gluconic Acid	+	ND	ND	+
D-Glucuronic Acid	+	ND	ND	–
D-Mannitol	+	+	+	+
D-Mannose	+	+	–	+
D-Serine	+	ND	ND	–
D-Sorbitol	+	+	+	–
D-Trehalose	+	+	–	+
D,L-α–Glycerol Phosphate	+	ND	ND	–
D,L-Lactic Acid	+	ND	ND	–
Glycerol	+	+	-	+
L-Aspartic Acid	+	ND	ND	–
Maltose	+	–	–	–
N-Acetyl D Galactosamine	+	ND	ND	–

N-Acetyl D Glucosamine	+	+	+	+
Pyruvic Acid Methyl Ester	+	ND	+	–
Succinic Acid	+	ND	–	–
Mono-Methyl Succinate	+	ND	ND	–
Sucrose	–	ND	–	–
